# Application of Response Surface Methodology for Optimization of Nanosized Zinc Oxide Synthesis Conditions by Electrospinning Technique

**DOI:** 10.3390/nano12101733

**Published:** 2022-05-18

**Authors:** Aizhan Rakhmanova, Sandugash Kalybekkyzy, Baktiyar Soltabayev, Aiman Bissenbay, Nazym Kassenova, Zhumabay Bakenov, Almagul Mentbayeva

**Affiliations:** 1 Department of Chemical and Materials Engineering, School of Engineering and Digital Sciences, Nazarbayev University, Nur-Sultan 010000, Kazakhstan; aizhan.rakhmanova@nu.edu.kz (A.R.); zbakenov@nu.edu.kz (Z.B.); 2 National Laboratory Astana, Nazarbayev University, Nur-Sultan 010000, Kazakhstan; baktiyar.soltabayev@nu.edu.kz (B.S.); aiman.bissenbay@nu.edu.kz (A.B.); nazym.kassenova@nu.edu.kz (N.K.)

**Keywords:** zinc oxide, electrospinning, optimization, response surface methodology

## Abstract

Zinc oxide (ZnO) is a well-known semiconductor material due to its excellent electrical, mechanical, and unique optical properties. ZnO nanoparticles are widely used for the industrial-scale manufacture of microelectronic and optoelectronic devices, including metal oxide semiconductor (MOS) gas sensors, light-emitting diodes, transistors, capacitors, and solar cells. This study proposes optimization of synthesis parameters of nanosized ZnO by the electrospinning technique. A Box–Behnken design (BB) has been applied using response surface methodology (RSM) to optimize the selected electrospinning and sintering conditions. The effects of the applied voltage, tip-to-collector distance, and annealing temperature on the size of ZnO particles were successfully investigated. Scanning electron microscopy (SEM) and transmission electron microscopy (TEM) images confirm the formation of polyvinylpyrrolidone-zinc acetate (PVP-ZnAc) fibers and nanostructured ZnO after annealing. X-ray diffraction (XRD) patterns indicate a pure phase of the hexagonal structure of ZnO with high crystallinity. Minimal-sized ZnO nanoparticles were synthesized at a constant applied potential of 16 kV, with a distance between collector and nozzle of 12 cm, flow rate of 1 mL/h, and calcination temperature of 600 °C. The results suggest that nanosized ZnO with precise control of size and morphology can be fabricated by varying electrospinning conditions, precursor solution concentration, and sintering temperature.

## 1. Introduction

Zinc oxide (ZnO) is an eminent semiconductor material due to its excellent electrical, mechanical, and unique optical properties. ZnO has a wide direct band gap width (3.37 eV), a vast excitation binding energy (60 meV), and ultraviolet (UV) absorption ability at room temperature, as well as various distinctive characteristics, including high electron mobility and excellent transparency [1,2,3]. It is well known that particle size reduction significantly affects the fundamental properties of semiconductor materials, especially nanosized metal oxides, which are typically 1–100 nm in size [4]. Nanosized metal oxides and semiconductors are of particular interest because they exhibit physical and chemical features and benefits [5,6], where their structural properties, lattice symmetry, and cell parameters as well as electrical and optical characteristics vary depending on their particle size [7,8].

In particular, ZnO nanoparticles are commonly used for the industrial manufacturing of microelectronic and optoelectronic devices, including metal oxide semiconductor (MOS) gas sensors, light-emitting diodes, transistors, capacitors, and solar cells [9,10,11]. The high surface area and large volume-to-diameter ratios of nanostructured ZnO (nanorods, nanoflowers, nanowires, and nanoparticles) have enticed scientists to expand their applications in various fields [12]. For instance, the photoactivity properties of ZnO are affected by particle size and surface-to-volume ratio, and nanometer-sized particles have a substantially higher photodegradation efficiency than micrometer-sized ones [13]. It was discovered that nanostructured ZnO gas sensors are chemically and thermally stable and able to operate at room temperature without heating, which helps with saving energy [14,15,16,17].

Several methods exist to synthesize ZnO, such as chemical vapor deposition, thermal evaporation, sol-gel, magnetron, and electrospinning [18]. Among them, electrospinning is the most effective, low-cost, versatile, and facile technique for the fabrication of nanofibers and nanoparticles with controllable size and morphology, using precursor solutions containing metal salts and polymers [19]. High voltage is applied to the precursor solution droplet during electrospinning, forming a Taylor cone. As a result, a nonwoven fiber mat is produced and collected on a substrate [18,20]. Many parameters influence the conversion of precursor solution to nanofibers, as well as the shape and diameter of the nanofibers. The set of properties associated with the spinning solution includes the polymer molecular weight, solution viscosity (controllable by solution concentration), surface tension, and solvent dielectric constant. Furthermore, process parameters include applied voltage, humidity, air velocity in the electrospinning chamber, temperature, the distance between the tip of the nozzle and the collector plate, flow rate of the solution, and collector rotation speed [21]. Electrospun metal oxide fibers are beneficial for producing self-assembled and self-arranged metal oxide particles with well-defined morphology and structure by annealing [22]. PVP-ZnAc nanofibers with a smooth surface and a diameter of 250 nm were also obtained by this method, followed by hydrothermal growth and thermal treatment. The diameter of fibers was minimized to 60 nm after the oxidation process, which was caused by the crystallization of ZnO and polymer decomposition [23,24,25]. With a similar technique, ZnO nanofibers that possess excellent photocatalytic activity were fabricated, and the effects of polymer concentration, annealing temperature, and time on the size and morphology were explored [26,27,28]. Likewise, several works have been reported on the synthesis of ZnO nanoparticles by using other polymers such as Polyacrylonitrile (PAN), polyvinyl acetate (PVA), and others. For instance, ZnO nanofibers with a diameter of 50–100 nm were formed by preparing PVA/ZnAc composite fibers using the electrospinning technique followed by annealing treatment [29]. It should be noted that synthesis parameters of ZnO nanostructured materials can be optimized by varying the solvent and precursor type, electrospinning parameters, and the effect of sintering temperature and environment [27].

To obtain ZnO nanoparticles of the required size and structure using electrospinning, it is essential to optimize the synthesis conditions by employing the response surface methodology (RSM) and the Box–Behnken design (BB). The main goal of RSM is to determine the optimal operating parameters of the system, and one of its advantages is the reduction in the number of experimental runs required to obtain sufficient data for acceptable statistical results. In addition, RSM makes it possible to evaluate the simultaneous interaction of these variables and model the selected response parameters; likewise, the predicted data is usually consistent with the experimental data [30]. In recent years, due to these benefits, RSM has become the most popular method for optimization processes [31,32,33]. Agarwal et al. [34] produced polylactic acid-based nanofibers and investigated the impact of various electrospinning conditions using the RSM method. It has been indicated that the distance between the collector and the nozzle, the applied voltage, and the concentration of the solution affect the diameter of the generated fibers. It was observed that the size of the fibers was first reduced and then increased with the increase of voltage, and these findings were consistent with the RSM method. Using the RSM method, the effective parameters on the diameter of polyurethane/polypyrrole-p-toluene sulfonate (PU/PPy-pTS) electrospun fibers were also studied, and the concentration of polymer solution and applied potential were found to be the most effective factor in determining of the fiber diameter [35]. Nowadays, RSM has been frequently utilized in the evaluation of the electrospinning conditions for fiber and particle synthesis [36,37,38], where statistical software Design Expert can be used for designing these experiments. So far, there has been no literature found on the optimization of ZnO nanoparticle-obtaining conditions by RSM in the electrospinning process using PVP and ZnAc. In this study, ZnO nanoparticles were synthesized from a PVP/ZnAc precursor solution using the electrospinning method and sintering, and their synthesis parameters were optimized by utilizing the RSM method. The use of RSM to optimize the conditions for electrospinning and sintering of ZnO nanoparticles facilitates the procedure and allows for the consideration of interactions between the selected parameters as well as the advancement of the process. Furthermore, guiding the experimental approach to save time and lower the cost assists in the control of morphology and diameter by conducting a smaller number of experimental runs. In addition, RSM helps evaluate the impact of each factor on the resulting ZnO diameter through a high-quality process [39,40].

PVP/ZnAc fibers were previously electrospun with fixed parameters such as precursor solution, annealing temperature, and spinning time to obtain a uniform diameter [41]. In this study, an efficient investigation of ZnO nanoparticle synthesis was carried out by optimizing and varying the parameters such as applied voltage and distance between collector and nozzle, sintering temperature, taking into account their interaction and effect of each condition. The modeling was conducted using Design Expert Software and the RSM method. Ideal conditions were set, under which pure and well-shaped ZnO nanoparticles with higher crystallinity and an average minimal diameter of 43 nm were synthesized by electrospinning and sintering processes. In our study, a power of 16 kV, a distance of 12 cm from the nozzle to the collector, and a sintering temperature of 600 °C were found to be optimal conditions for fabricating the smallest particles.

## 2. Materials and Methods

### 2.1. Materials

Materials for fiber preparation: Zinc acetate (ZnAc) (99.99% purity) and Polyvinylpyrrolidone (PVP, >99.99% purity, M_w_ = 40,000) were purchased from Sigma Aldrich (Amsterdam, The Netherlands). Al foil (~20 mm) and 99% pure ethanol were used.

### 2.2. ZnAc-PVP Nanofiber Preparation and ZnO Synthesis

PVP solution in ethanol (8 wt%) was prepared and stirred for 5 h. Then, ZnAc (1 g) was added to 10 mL of PVP solution and stirred for an additional 5 h. The spinning solution was injected through a stainless steel needle connected to a high-voltage DC power supply of 12–16 kV and a distance of 8–12 cm and electrospun. Nanofibers were accumulated on the Al foil, and the obtained PVP-ZnAc composite nanofibers were annealed in air at 600–800 °C (at a heating rate of 5 °C/min) for 2 h.

### 2.3. Experimental Design, Statistical Analysis, and Optimization by RSM

In the electrospinning and sintering processes, the most effective synthesis parameters and their ranges that affect ZnO particle diameter and properties were determined by performing a preliminary experiment and reviewing the literature [42]. These parameters were the applied potential (X_1_: 12–16 kV), the distance between the collector and the nozzle (X_2_: 8–12 cm), and the calcination temperature (X_3_: 600–800 °C). Fifteen experiments were conducted based on the BB design and optimized by the RSM method. Each experiment was run twice, and the average response was taken into consideration. The ranges of minimum and maximum coded values of process parameters in the present study were fixed according to the initial trial runs (presented in Table 1). A second-order polynomial model with the coded independent variables (X_i_,_j_) was used to obtain minimized-size ZnO particles (Y) as shown in the equation below (Equation (1)):

(1) $$Y={\;b}_{0}\overset{n}{\underset{i=1}{\sum }}b_{i}X_{i}+\overset{n}{\underset{i=1}{\sum }}b_{\mathrm{ii}}X_{i}^{2}+\overset{n}{\underset{i<1=1}{\sum }}b_{\mathrm{ij}}X_{i}X_{j}$$

Here, Y is the response variable to be modeled (ZnO size), X_i_ and X_j_ define the independent variables, b_0_ is the constant coefficient; b_i_ is the coefficient of linear effect, b_ij_ is the coefficient of interaction effect, b_ii_ is the coefficient of quadratic effect, and n is the number of variables. To specify the significance of the model, an ANOVA (analysis of variance) was conducted. The response surface and contour plots of the model-predicted responses were applied to specify the interactive relations between the significant variables. Design Expert, v. 8.0.7.1 (Stat-Ease Inc., Minneapolis, MN, USA), was used for designing the tests as well as regression and graphical analysis of the obtained data.

### 2.4. Characterization

The microstructure and morphology of the fibers and ZnO nanoparticles were observed by scanning electron microscopy (SEM, EDX ZEISS Crossbeam 540, Zeiss, Germany). Samples for SEM analysis were coated with 5 nm gold by an automatic sputter coater (Q150T, Tokyo, Japan) to reduce charging. The structure of the obtained fibers and ZnO nanoparticles were observed by transmission electron microscopy (TEM, JEOL JEM-1400 Plus, Peabody, MA, USA). The accelerated voltage of the TEM was 120 kV. The structural properties of the ZnO samples were characterized by X-ray diffraction (XRD; Rigaku SmartLab, Tokyo, Japan). The optical properties of the obtained samples were studied using an Evolution 300 UV-Visible Spectrophotometer.

## 3. Results and Discussions

### 3.1. Response Surface Model

**Figure 1:**
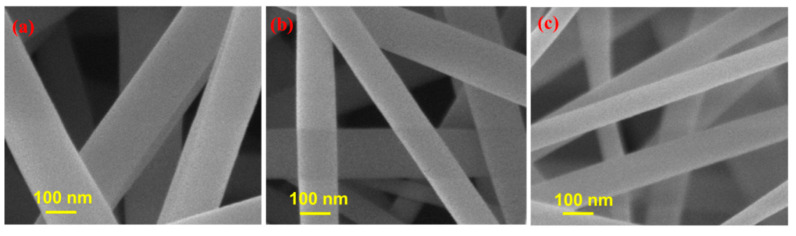
SEM micrographs of the PVP-ZnAc fibers at a constant distance of 12 cm; a flow rate of 1 mL/h; and applied potential of (**a**) 12 kV, (**b**) 14 kV, and (**c**) 16 kV.

This study used an efficient RSM modeling approach based on a three-level BB design with three variables to reveal the influence of the chosen spinning and sintering parameters on the size of the electrospun and sintered ZnO nanoparticles. Optimization is highly desired to see the effect of the various electrospinning conditions on the structural integrity of fibers. To achieve optimal conditions, a set of measurable investigative factors such as applied potential, distance between nozzle and collector, sintering temperature, and the observed response of the specified conditions were selected. Among the chosen parameters, the most influential one is the applied potential because it directly causes elongation of a polymer fluid drop retained at a needle tip by surface tension. At some threshold potential difference, the charge repulsion overcomes surface tension, and viscous forces within the fluid drop. A complex force balance governs the ejection of a fluid jet and the subsequent creation of nanofibers [20]. It has been noted that the formation of electrical arcs between electrodes is responsible for the discontinuous withdrawal of fluid jet beyond the maximum potential difference and the smallest separation distance set [43]. In the current research, the potential difference was divided into three levels, ranging from 12 kV to 16 kV. The spinning of the fibers started at 12 kV, and an accumulation of polymer solution mass near the needle tip, combined with the short residence time of the droplets, can explain the erratic behavior of the solution. The fibers obtained at this voltage in the observed microscopic fields had larger diameters. Bead-free weaved threads were electrospun under a constant feed rate (1 mL/h) and separation distance value (12 cm) with an applied potential difference of 12 kV (Figure 1a), where the average size of the obtained fibers was 193 nm; 14 kV (Figure 1b), where the average size of the obtained fibers was 125 nm; and 16 kV (Figure 1c), where the average size of the obtained fibers was 91 nm. Drop formation at the needle tip should have a longer residence time, and the charged ejected jet should have a longer “time-of-flight” under these ideal conditions, which should boost solvent evaporation and the formation of fiber from the solution [44].

**Figure 2:**
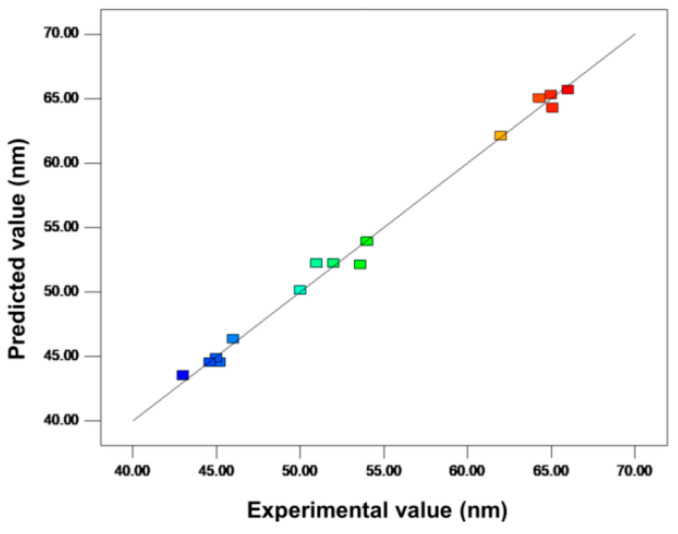
Plot of the model-predicted versus observed response of ZnO particle average size.

Three variables of the spinning—calcination temperature (X_1_), tip-to-collector distance (X_2_), and applied potential (X_3_)—were selected for the optimization process to form ZnO particles. The interaction effect of the chosen parameters on the response observed in the experimental runs can be explained using the analysis of variance (ANOVA). Furthermore, the adequacy of the model was examined using diagnostic diagrams, and the model should be validated by evaluating the optimum experimental conditions, as previously explained [45]. The optimization results show the parameter interaction effect, which consists of 15 experiments, as is presented in Table 2. For each experiment, the ZnO diameter (the response) was measured; a two-set average is noted in Table 3, column Y. When one decides if the overall results are significant, the F statistic must be used in combination with the *p*-value. The *p*-value indicates the degree to which the data is consistent with the null hypothesis. The successive *p*-value of <0.0001 and F value of 99.22 indicate significant model terms. In this scenario, there were key model terms: A, B, C, A^2^, B^2^, and C^2^. Only the quadratic terms for the two electrospinning variables made it into the refined model, which was an interesting finding [43]. Coefficient of variance (CV) indicates the reproducibility of the model, for which a value of less than 10% is desirable [46]. According to the results, the CV value is 3.38%, and the model was statistically valid. The predicted residual error sum of squares (PRESS) is cross validation used in data analysis to offer a statistical summary of model performance [47]; the obtained PRESS of the model is 58.71. The adjusted *R*^2^ of 0.9546 in the improved model agreed reasonably well with the expected *R*^2^ of 0.9424. Figure 2 depicts the relationship between the measured diameter of the ZnO particles and the models’ anticipated diameter. The linear correlation coefficient indicates reasonable agreement between the experimental and model values across the factor space.

### 3.2. Response Surface Plots

**Figure 3:**
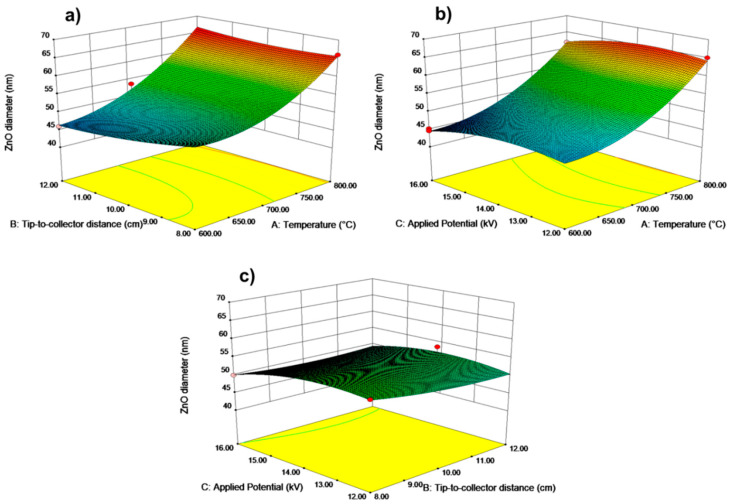
Response surface plots of ZnO diameter (response) versus (**a**) distance and temperature, (**b**) voltage and temperature, and (**c**) voltage and distance.

The ZnO nanoparticles’ diameter and the response variables are shown in three-dimensional surface plots in Figure 3 versus two factors at a time (with the third variable kept constant at the center value of (0)). As shown in Figure 3b, at 16 kV applied potential and 12 cm distance (feed rate of 1 mL/h constant), nanoparticles with the smallest diameter were formed. However, when the voltage and distance were reduced to the absolute minimum of 12 kV and 8 cm, the largest average diameter fibers (193 nm) were generated. These plots revealed a few generalized conclusions about electrospun ZnO nanoparticles: (a) the particle diameter changed in an inverse relationship to the applied voltage, (b) increasing the distance of separation led to a decrease in fiber diameter, and (c) fiber diameter was smaller for the minimal value of calcination temperature of 600 °C.

The Box–Behnken (BB) design [48] is a part of the standard response surface approach and can be used to establish, analyze, and identify the quantitative correlation among electrospinning variables and mean particle diameter. When compared to other symmetrical second-order experimental designs such as Doehlert, central composite, and three-level factorial designs in terms of efficiency and characteristics, the use of BB design is common in industrial research due to the economic advantages that require only three levels for each factor, with the settings of 1, 0, +1 [49]. In this study, the relationship between the following variables was investigated using the BB design three-level, three-factor model. In combination with RSM, this design is frequently used to optimize a variety of physical, chemical, and biological processes [50,51]. Fiber diameter accord of different polymeric materials has been studied using various electrospinning parameters [52]. Numerous studies on electrospinning support these findings of fiber diameter variation in response to parametric manipulation. Changing the distance between the tip and the collector which has a tremendous effect on flight time and field strength significantly impacts the fiber morphology. Separation distance and fiber diameter are opposite to each other, and in many cases, the droplet formation was due to the inability of the jet to maintain sufficient distance between the tip and the collector as a result of the increased field strength [53]. To stretch the solution before it deposits on the collector, one can increase the separation distance, leading to a decreased fiber diameter [54]. An increase in fiber diameter is associated with decreased field strength when separated by a greater distance [55]. However, fibers are not deposited if the separation distance is too large [54]. Because of this, it appears that voltage and the resulting electric field have a significant impact on the jets’ stretching, acceleration, and diameter. A higher voltage causes the solution to be stretched out more in the jet due to stronger columbic forces, which reduces the fiber size [56,57]. It is known that the formation of beads cannot be completely avoided at higher voltages. However, here, no beads were observed in the selected range of applied potential, and it was excellent for the fabrication of nanosized fibers. Below 12 and above 16 kV, the possibility of the formation of beads is raised. The density of the beads increases with the rise of voltage, and the beads unite to form a thicker diameter fiber [50,51,58].

### 3.3. Response Surface Plotting and Characterization of ZnO Nanoparticles at Optimized Conditions

**Figure 4:**
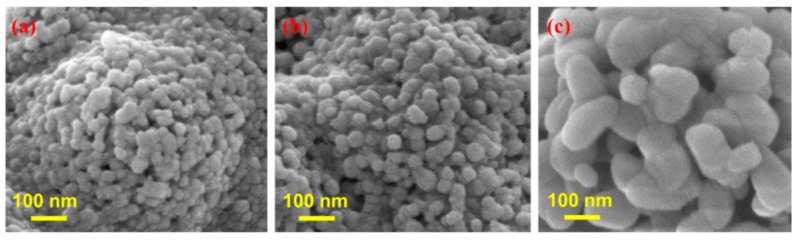
SEM images of the ZnO nanoparticles synthesized at a constant applied potential of 16 kV; a distance between collector and nozzle of 12 cm; a flow rate of 1 mL/h; and calcination temperature of (**a**) 600 °C, (**b**) 700 °C, and (**c**) 800 °C.

**Figure 5:**
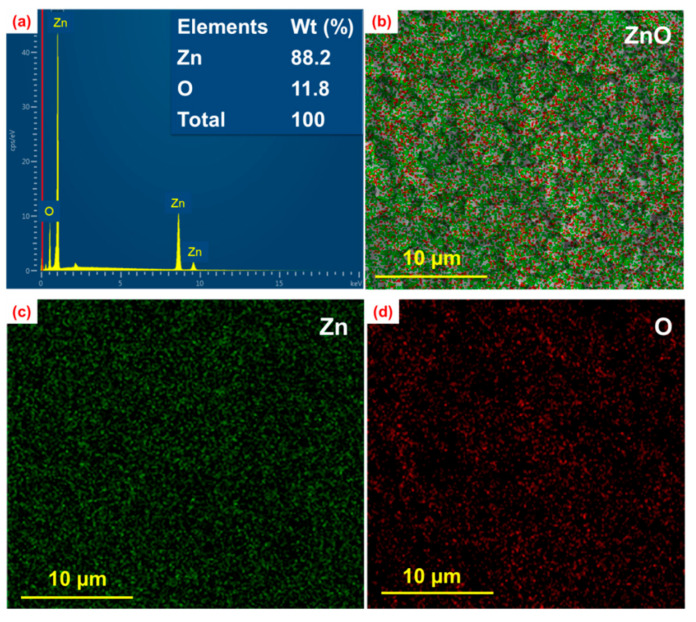
(**a**,**b**) EDS mapping showing the distribution of ZnO nanoparticles, (**c**) zinc, and (**d**) oxygen synthesized at an applied potential of 16 kV, a distance of 12 cm, a flow rate of 1 mL/h, and a calcination temperature of 600 °C.

As demonstrated in Figure 3, response surface plots illustrate the effect of interaction between sintering temperature, the distance between nozzle and collector, and the applied voltage on the size of ZnO nanoparticles. The nanoparticle size became bigger as the calcination temperature increased from 600 to 800 °C. The fundamental reason for this is that an increase in temperature influences the growth of crystal. The particle size grew rapidly as the temperature rose from 600 to 800 °C. These findings could imply that the increase in size was mainly due to crystal development, as evidenced by multiple previous investigations [59,60]. When these two parameters (voltage and calcination temperatures) are combined, they have a more significant impact on particle size, comparable to the effects seen in Figure 3a,b. At a constant calcination temperature of 600 °C, the particle size of ZnO changes dramatically as the voltage is reduced from 16 to 12 kV. The electrospinning process and calcination temperature highly influenced the diameter of ZnO nanoparticles. Previous optimization by employing response surface methodology was used to form smooth and homogeneous nanofiber architectures [59]. SEM images shown in Figure 1 represent fibers before annealing, whilst Figure 4 shows those after calcination. Figure 5 provides EDS mapping of the obtained ZnO nanoparticles. By optimizing the electrospinning parameters, uniform and bead-free fibers were obtained. The average diameter of electrospun and sintered ZnO nanoparticles was determined using the Image J program. The experimental design parameters were evaluated by varying processing settings with a fixed collector distance of 12 cm and applied potential in the range from 12 kV to 16 kV. A significant decrease in diameter is due to the fact that a higher electrical voltage and distance contribute to a greater stretching of the polymer, resulting in a decrease in the diameter of the electrospun fiber and particles. A constant flow rate of 1 L/min was maintained throughout the experiment. As shown in Figure 4a–c, the formation of well-shaped ZnO particles can be observed after annealing of the obtained fibers at various temperatures from 600 °C to 800 °C.

We performed an analysis of SEM/EDS micrographs of all electrospun ZnO nanoparticles shown in Figure 5. From the EDS report, the weight percentage and atomicity of Zn and O were 88.2 and 11.8, respectively, which is close to the bulk ZnO weight percentage.

**Figure 6:**
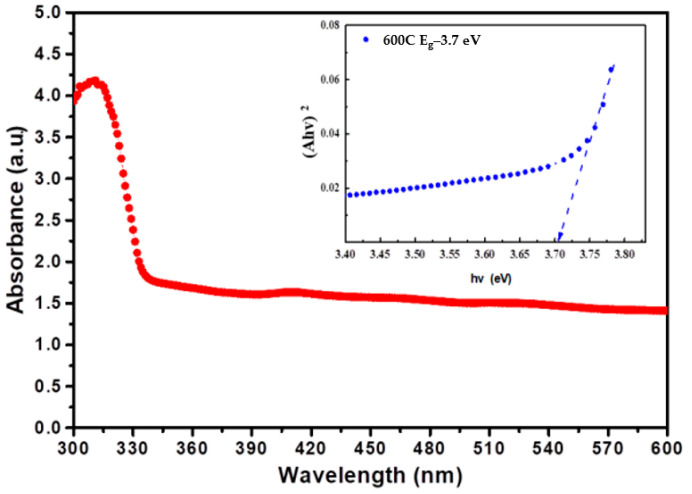
UV-Visible spectra and energy band gap of ZnO nanoparticles synthesized at a constant applied potential of 16 kV, a distance between collector and nozzle of 12 cm, a flow rate of 1 mL/h, and a calcination temperature of 600 °C.

Figure 6 displays the UV-Vis absorption spectra of ZnO nanoparticles produced at optimized conditions using the electrospinning technique; absorbance was measured in the range of 300–600 nm wavelengths. With the decrease of ZnO particle size, the energy gap widens due to their quantum size effect, caused by the confinement of electrons within particles of dimensions smaller than the bulk counterpart [61]. The energy band gap of a semiconductor becomes more pronounced when the size of the nano-crystallites is smaller than the Bohr radius of the bulk excitation [62]. In nanoscale materials, columbic interactions between holes and electrons are critical and charge carriers can be quantum bound, which changes the semiconductor’s valence and conduction bands [63].

The UV-Vis absorption spectra of synthesized ZnO nanoparticles showed an absorption peak at 320 nm. The Tauc plot of the samples showed that the band gap of the ZnO nanoparticles prepared at a calcination temperature of 600 °C was 3.72 eV, which is comparable to the previously reported value [64]. Equation (2) was used to calculate the direct band gap energy:

(2) $$\alpha h\upsilon =A{\left[h\upsilon -E_{g}\right]}^{n}$$

Characterization of ZnO nanoparticles using UV-Vis spectrum analysis is usually used to study the size and shape of the particles [65]. The rate and width of the surface plasma on the absorbent depend on the size and shape of the nanoparticles. It is worth noting that the obtained E_g_ value was different from the band gap of bulk ZnO (3.37 eV). Bang gap can be attributed to the optical confinement effect, corresponding to the size and length of nanoparticles [66].

### 3.4. XRD Patterns and Transmission Electron Microscopy (TEM)

**Figure 7:**
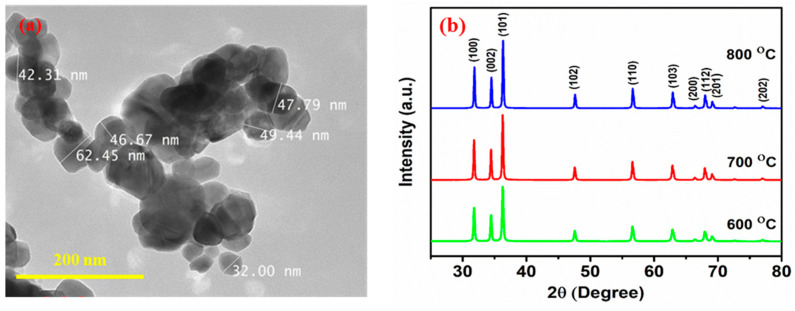
(**a**) TEM micrographs of ZnO nanoparticles synthesized at a constant applied potential of 16 kV, a distance between collector and nozzle of 12 cm, a flow rate of 1 mL/h, and a calcination temperature of 600 °C. (**b**) XRD patterns of ZnO nanoparticles synthesized at a constant applied potential of 16 kV; a distance between collector and nozzle of 12 cm; a flow rate of 1 mL/h; and calcination temperatures of 600 °C, 700 °C and 800 °C.

TEM examination was conducted to ascertain the size of the nanoparticles (given in Figure 7a). The ZnO nanoparticles fabricated under optimized conditions reveal that the particles are hexagonal with slight variation in thickness, which corroborates with the SEM results. The majority of ZnO nanoparticles are sphere-shaped and have an average particle size of 43 nm. The XRD patterns of ZnO nanoparticles fabricated by electrospinning at calcination temperatures of 600, 700, and 800 °C are shown in Figure 7b. All of the peaks in the XRD patterns correspond to the structure of ZnO wurtzite. The intensity of the peaks and the average crystallite size increased with the increasing calcination temperature, indicating the formation of ZnO nanoparticles with a larger size and an increase in crystallinity [67]. The diffraction peaks were assigned to the ZnO (100), (002), (101), (102), (110), (103), (200), (112), (201), and (202) planes, respectively. All of the peaks were in excellent agreement with the JCPDS card No. 89-1397, indicating the development of pure ZnO phase upon calcination [41].

## 4. Conclusions

The present work vouches for the successful application of a BB design and RSM method to predict the diameter of electrospun and sintered ZnO nanoparticles. The selected method and the applied experimental design effectively determined the optimal parameters of the three chosen variables to fabricate ZnO nanoparticles with minimal diameter by the electrospinning technique. The selected variables (applied potential, distance between nozzle and collector, and calcination temperature) had a significant effect on the ZnO nanoparticles’ size, where the results of a second-order polynomial regression model were satisfactory. The model can be used to develop strategies for fine tuning the shape and size of electrospun fibers and particles depending on the application. In this work, ZnO nanoparticles with an average size of 43 nm were successfully synthesized under optimal conditions. The optimal electrospinning conditions to produce minimum-size ZnO nanoparticles were an applied potential of 16 kV and a distance between the collector and nozzle of 12 cm. To obtain pure and hexagonal structure ZnO nanoparticles from the ZnAc-PVP fiber, a calcination temperature at 600 °C was found to be optimum. SEM, EDS, and TEM characterization results describe the formation of well-shaped ZnO nanoparticles; the XRD pattern indicates a highly crystalline pure phase and that the band gap of the ZnO nanoparticles is 3.72 eV.

## Figures and Tables

**Table 1:** Parameters and levels for the optimal design.

Independent Variables	Factor X_i_	Range and Level		
		−1	0	+1
Applied potential (kV)	X_1_	12	14	16
Distance (cm)	X_2_	8	10	12
Calcination temperature (°C)	X_3_	600	700	800

**Table 2:** ANOVA for response surface reduced quadratic model.

Source	Sum of Squares	df	Mean Square	F Value	*p*-Value
Model	982.28	3	327.43	99.22	0.0001 significant
A-Temperature	593.77	1	593.77	179.93	0.0001
B-Distance	0.093	1	0.093	0028	0.8697
C-Applied potential	9.19	1	9.19	2.79	0.1233
Residual	36.30	11	3.30		
Lack of fit	35.62	9	3.96	11.64	0.0816 not significant
Pure error	0.68	2	0.34		
Cor total	1018.58	14			
Std. Dev.	1.82		*R* ^2^	0.9644	
Mean	53.79		Adj *R*^2^	0.9546	
CV %	3.38		Pred R-Square	0.9424	
PRESS	58.71		Adequate Precision	22.609	

**Table 3:** Box–Behnken design matrix containing 15 experimental runs.

Run Order	Calcination Temperature (°C)	Tip-to-Collector Distance (cm)	Applied Potential (kV)	Response, Y (ZnO Size in nm)	Predicted Values (ZnO Size in nm)
1	800	8.00	14.00	66	65.68
2	800	8.00	12.00	65	65.30
14	600	10.00	16.00	46	46.34
9	700	8.00	12.00	64.30	65.03
8	800	10.00	16.00	45.20	44.52
10	600	12.00	12.00	65.10	64.28
5	600	8.00	16.00	53.60	52.12
6	800	10.00	12.00	62.00	62.11
7	700	12.00	14.00	54.00	53.90
11	700	8.00	16.00	45.00	44.88
15	700	10.00	14.00	50.00	50.12
3	600	12.00	14.00	44.00	44.50
13	700	10.00	14.00	52.00	52.23
4	800	12.00	12.00	44.60	44.52
12	600	12.00	16.00	43.00	43.23

## Data Availability

All data supporting the conclusions of this article are included within the article.
